# A Computational Simulation Study of Benzamidine Derivatives Binding to Arginine-Specific Gingipain (HRgpA) from Periodontopathogen *Porphyromonas gingivalis*

**DOI:** 10.3390/ijms11093252

**Published:** 2010-09-13

**Authors:** Dooil Kim, Dae-Sil Lee

**Affiliations:** Systems Microbiology Research Center, KRIBB, Daejeon, 305-806, Korea

**Keywords:** Porphyromonas gingivalis, Arg-gingipain, free energy, molecular dynamics

## Abstract

We have shown that the binding free energy calculation from molecular dynamics can be adapted successfully to cysteine proteinases, such as arginine-specific gingipain (HRgpA) from *Porphyromonas gingivalis*. The binding free energy obtained is in good agreement with the available experimental data for eight benzamidine derivatives including urea and ether linker. The calculations showed that the electrostatic energies between HRgpA and inhibitors were important in determining the relative affinities of the inhibitors to the HRgpA, with an average binding free energy of about −5 kcal/mol. The average structures of the eight complexes suggest that benzamidine inhibitors interact with Asp387, His435, and Cys468 by hydrogen bonding and with Trp508 by hydrophilic interactions that are essential for the activities of benzamidine inhibitors. It can therefore be expected that the method provides a reliable tool for the investigation of new HRgpA inhibitors. This finding could significantly benefit the future design of HRgpA inhibitors.

## 1. Introduction

The periodontal ligament derived from connective tissue is continuously turned over in a tightly controlled cycle, which is consistently challenged by invasion of pathogenic bacteria, such as *Porphyromonas gingivalis*. They can accumulate on the gum surface and induce an inflammatory response from the host tissue. This immune response, which serves to destroy any foreign bodies, may upset homeostasis within the periodontium and lead to gingivitis [1–6].

Gingipains are one of the virulence factors that cause the development of periodontitis and encourage the excretion of trypsin-like cysteine proteinases from the well established oral pathogen *Porphyromonas gingivalis*, a gram negative anaerobic rod, and a major causative bacterium of adult periodontitis. There are three major gingipains: Gingipain K (Kgp), which cleaves exclusively on the C-terminal side of lysine residues, and two gingipain R types (HRgpA and RgpB), which are specific for Arg-Xaa peptide bonds. HRgpA and Kgp have catalytic and adhesion/hemagglutinin domains connected by non-colvalent complexes, but RgpB has only a catalytic domain with a primary structure [7–13].

The design of irreversible inhibitors for HRgpA generally involves a peptide chain with a so-called warhead replacing the scissile peptide bond. The optimal peptide sequence for the inhibitor is derived from the best peptide substrate sequence, which can be determined through HRgpA subsite mapping using a peptide library. The length of the peptide portion also plays an important role in specificity. It is, therefore, possible to design inhibitors specific for HRgpA. The inhibitor can then be used to investigate the physiological significance of the HRgpA. The warhead contains a reactive functionality that is attacked by the HRgpA’s catalytic nucleophile. Thus far, several warheads for HRgpA have been developed. Once a peptide sequence and warhead are determined, a strategic structural analysis relationship (SAR) study is performed to optimize the parent compound and its inhibitory potency. Since the gingipains play a central role in the pathogenesis of gingivitis and periodontal disease, justification exists for developing potent inhibitors as potential therapeutics.

In the present study, the Poisson Boltzmann Solvent Accessible Surface Area (PB-SASA) method was used as a predictive tool to study HRgpA:inhibitor modes of interaction. Eight compounds with potent anti-periodontopathogenic activities [14] were selected for the current project. The experimental molecular structure of complexes formed between these inhibitors and HRgpA has not previously been reported. To determine the most likely model of interaction between the studied benzamidine derivatives (used as inhibitors) and HRgpA, the binding free energy was measured experimentally for each molecule interacting with HRgpA; in parallel, binding free energies were calculated using the PBSASA method for different topological models of HRgpA:inhibitor complexes. A comparison of the experimental free energy data with calculated values for the different binding models enabled us to propose the most likely mode of interaction of each studied inhibitor with HRgpA.

## 2. Results and Discussion

The binding of eight benzamidine analog inhibitors (Table 1) to the *Porphyromonas gingivalis* gingipain R (HRgpA), which is the non-covalent complex of the catalytic domain with hemagglutinin/adhesion domains derived from the C-terminal extension, was investigated using automated docking and binding free energy calculations from molecular dynamics simulations. This enables a detailed structural analysis of binding modes and the identification of the key inhibitor-HRgpA interactions that contribute to the free energy of binding. The simulated compounds are a subset of the inhibitors for which experimental binding affinities have been published by Krauser *et al.* [14], and they were chosen as they include both the least and most potent inhibitors from that series, and they display a fairly wide variety of structural features. All inhibitors share a common benzamidine moiety, with substituents consisting of aromatic ring derivatives by urea and ether linkers.

### 2.1. Homology Modeling

**Figure 1:**
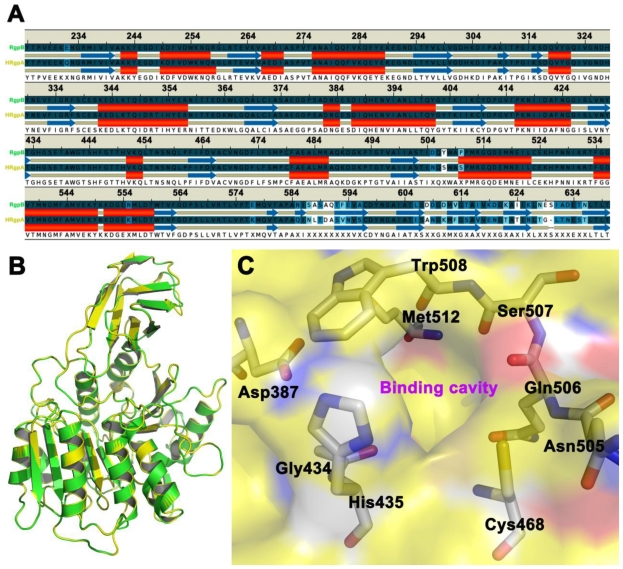
Panel A shows the sequence alignment of the amino acid sequences of the selected region of HRgpA and the secondary structure alignment. Panel B shows the three-dimensional homology model of HRgpA (yellow color) superimposed on the RgpB (green color) crystal structure [15] that was used as a template. The color coding follows that of the gene name in Panel A. The side chains of the residues proposed to be involved in inhibitor binding are shown as sticks in Panel C.

The homology model of HRgpA is more or less structurally identical to the active sites region of the crystal structure of gingipain R (RgpB) with a single chain catalytic domain (PDB accession code 1CVR [15]) with high sequence identity. For the residues that form the inhibitor binding cavity, and thus can be expected to be important for correct docking and molecular mechanics interactions, the root mean-square deviation (RMSD) of the HRgpA model compared to the RgpB crystal structure is 0.18 Å when a theoretical model was superimposed on the crystal structure (Figure 1). It also should be emphasized here that HRgpA and the template RgpB have identical sequences in the cavity region, such that the problem of arbitrarily modeling initial (before molecular dynamics) sidechain rotamers is not an issue in this case.

### 2.2. Automated Docking

**Figure 2:**
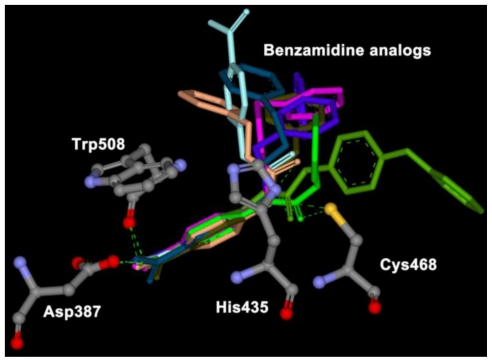
Representative binding poses of eight benzamidine analogs in core binding sites given by the docking calculation.

Each docking simulation generated 100 docked conformations of each inhibitor. The docking procedure generally generated slightly more diverse docking poses for the inhibitors containing urea or ether linkers. Encouragingly, out of these 800 poses generated, only a handful fall outside of a consensus orientation in which the benzamidine part of the inhibitor is positioned in the binding cavity of the HRgpA, which is in close proximity to Asp387, His435, Cys468, and Trp508 (Figure 2). In these poses, one of the substituents (generally the largest one) points towards the outside of its cavity.

**Figure 3:**
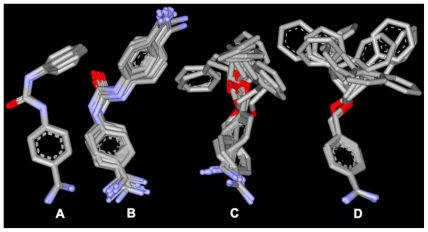
Top 10 docking solutions for inhibitors: (**A**) Bz1; (**B**) Bz4; (**C**) Bz6 and (**D**) Bz7. The average heavy atom RMSD relative to the top-ranked pose is less than 3.0 Å.

Of the top 10 ranking solutions for any given inhibitor, taking only heavy atoms into account, the average RMSD compared to that of the top-ranked solution was approximately 2.0 Å, with few poses deviating >3.0 Å from the top-ranked pose. Typical results are shown for inhibitor Bz7 in Figure 3. Conformations that deviated more did so because the orientation of the substituents was flipped with respect to the other poses; however, the benzamidine part remained in the same position. Even for inhibitor Bz6, which showed a relatively large diversity in the suggested binding poses, the positioning of the benzamidine part of the inhibitor is very well determined; the positions of the benzamidine nitrogens–for all but five poses (all of which are among the 10 lowest ranked)–are all situated within the van der Waals surface of the top-ranked pose. The smallest compounds, Bz1 and Bz4, showed the least deviation between docking poses. In this case, the heavy atoms of the top 20 docking poses were all within 1.8 Å RMSD of the highest-ranked pose, with a corresponding average RMSD of 1.5 Å.

### 2.3. Molecular Dynamics

**Figure 4:**
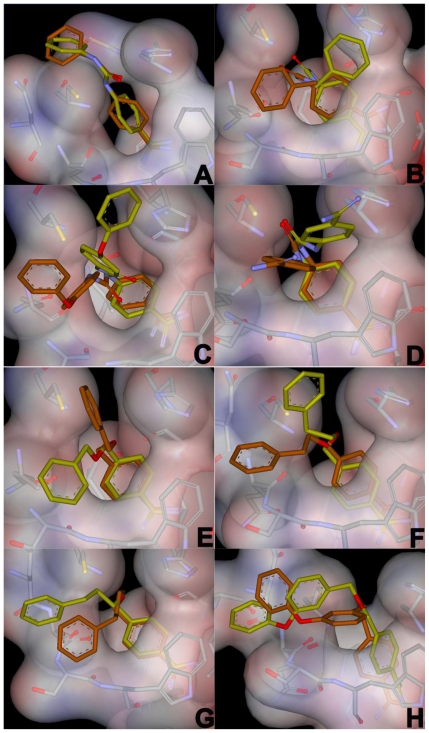
Comparison between the average structures from the production phase MD simulations in the bound state (yellow) and the corresponding docking poses (brown) used as starting conformations for the MD simulations. The average structures shown are from the simulations that yielded the lowest estimated free energy of binding.

For each inhibitor, the top five binding poses from the automated docking experiments were chosen for further investigation using molecular dynamics. Out of these 40 poses, all but one had the benzamidine part of the inhibitor positioned in the pivot region of the cavity. Generally, the inhibitor positions are stable during MD simulation, and the average structures from the production phase MD deviates relatively little from the docked positions. The heavy atom RMSD of the poses shown during MD simulation in Figure 4 are an average of approximately 2.5 Å more than their corresponding docked poses. The ether linkers have few or no specific interactions with the protein, and thus are fairly flexible during the MD simulations; whereas, the urea portions of the inhibitor are typically more stable in their positions, sometimes alternating between equivalent sites on the core cavity.

### 2.4. Binding Free Energy

**Figure 5:**
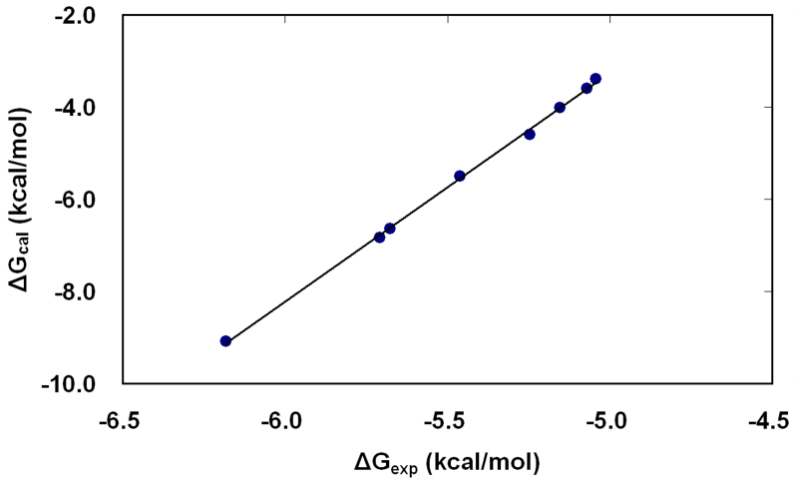
The correlation between the free energies of binding of inhibitors (Bz1–Bz8) as calculated by molecular dynamics in combination with the PB-SASA method (Δ*G*_cal_ from calculation) *versus* those derived from the experimental data of Krauser *et al.* (Δ*G*_exp_ from experiment) [14]. The lowest binding free energy estimate from the five different poses simulated for each inhibitor is plotted against the corresponding value calculated from experimentally determined *K_i_*-values.

The calculated values of HRgpA:inhibitors binding free energy for eight benzamidine derivatives are presented in Table 2. The binding free energy is dissected according to the approach outlined in the Methods section for electrostatic (Δ*G*_elec_) and non-electrostatic (Δ*G*_vdW_) contributions. The first one was calculated by solving the PB equations, the second by using the SASA method. The last column in Table 2 reports the total HRgpA:inhibitors binding free energy, which can be compared directly with the experimental values of binding free energy presented in Table 1. The contribution of the entropy term in our free energy calculations accounts for the reduction of translational and rotational freedom of an inhibitor and HRgpA upon binding. The correlation between the lowest calculated free energy of binding (obtained from PB-SASA and MD) for each inhibitor and the relative free energy differences derived from the experimentally determined kinetic constant values is very good (Figure 5). The ranking of the inhibitors also is in very good agreement with experiments.

Comparing different contributions to the binding free energy in Table 2, one can say that the driving forces for inhibitor binding and eventual binding onto HBgpA are electrostatic interactions resulting from the burial of interfacial surface area of both HBgpA and the inhibitor. The inhibitors, especially Bz06 and Bz07 with relatively long side chains, may be regarded as more flexible than other inhibitors. Therefore, one may assume somewhat higher values than were calculated for others. This entropy cost is due to the advantage of conformational chain freedom and is calculated to be in the order of approximately 1.3–2.2 kcal/mol for less flexible and short chains. Taking into account the entropy correction, we find that our calculated values of HRgpA:inhibitor binding free energy are very close to the experimental data.

Although the van der Waals interactions between the HRgpA residues of the core cavity and the inhibitors are relatively weak compared to electrostatic forces, they play a key role in determining the binding affinities of the inhibitors. In fact, ranking according to Δ*G*_vdW_ is identical to the ranking according to Δ*G*_bind_, with the exception of compound Bz7. This inhibitor also has a significantly less hydrophilic substituent than the other compounds, and the electrostatic interactions may thus be expected to contribute more to binding. Such that, although the electrostatic or polar interactions give a larger overall contribution to the calculated binding free energies, that contribution is relatively uniform for the different inhibitors as compared to non-polar interactions. In contrast, the relatively large differences in the non-polar contributions are more important in determining the relative potency of the inhibitors.

The compound with the highest calculated binding affinity (Bz4) has a strong electrostatic interaction with the active sites of HBgpA and water in the bound state. The electrostatic contribution for the predicted model of binding Bz4 is more negative than others. This means that protonated chains of Bz4 prefer to stay in the HBgpA subsite and in bulk solvent equally. From this point of view, the relatively high calculated binding affinity for compound Bz4 is surprising, given its structural similarity to Bz1 and Bz5 and the similar binding poses suggested by the docking procedure. Indeed, based solely on the differences in structure between Bz1, Bz4, and Bz5, one might even suggest that given the nature of the binding site, Bz4 would be expected to be the best inhibitor of the three. Since Bz1 and Bz5 are identical, except for the urea linker in Bz1 and the ester linker in Bz5, and since the binding site is a highly hydrophilic cavity, the less polarized ester linker would be less sensitive to solvation and the binding site than the urea linker.

If only inhibitor-water interactions are taken into account, a clear picture emerges in which the five benzyl-containing compounds (Bz1, Bz2, Bz5, Bz6, and Bz7) lose energy in electrostatic interactions with water when transitioning from the free state to the bound state; whereas, the rest of the inhibitors have roughly as strong polar interactions with water in both the free and the bound states.

## 3. Computational Methods

### 3.1. Homology Modeling and Docking

The generation of a three-dimension homology model of gingipain R type A (HRgpA) from porphyromonas gingivalis using MODELLER (www.salilab.org) was based on the complex crystal structure of gingipain R type B (RgpB), using its peptidyl inhibitor (PDB accession code 1CVR [15]) as a template. Only the catalytic domain (residues 225–652, HRgpA numbering, which is the catalytic domain) of HRgpA was included in the model. In this region, the sequence similarity between RgpB and HRgpA is approximately 90%, and the sequences align without any gaps at all, which allows the construction of a very reliable homology model. Next, the system was prepared for docking and molecular dynamics (MD) simulations.

All the benzamidine derivatives that were used as inhibitors were energy-minimized with the MMFF force field [16] before docking. Automated docking of the inhibitors was performed using the flexible docking (FD) module [17] in Discovery Studio 2.0 (Accelrys Inc.). To predict optimized binding poses for flexible inhibitors in a flexible binding site of HRgpA, the FD module was used in combination with ChiFlex to generate side chain conformations of HRgpA; CatConf/CAESAR was used for building diverse low energy conformations of inhibitors; LibDock was used for computing hotspot locations; ChiRotor was used for modifying side chain conformations of HRgpA; and CDOCKER was used to determine annealing and minimizing inhibitor poses. The active site radius was set to 7.0 Å, centered near a point on the symmetry axis of the center of mass (CM) of the binding cavity. This positioning of the docking sphere enables the inhibitors to explore different docking conformations within the entire cavity. LigandFit [18] was then set to terminate the docking if the top three poses for an inhibitor were within 1.5 Å RMSD. The docking procedure was performed, and the complexes were again solvated for molecular dynamics and free energy calculations.

### 3.2. Molecular Dynamics Simulation

All MD calculations were conducted using the program AMBER with the modified all-hydrogen AMBER parameter set [19–22]. Partial atomic charges were assigned to the inhibitors, analogous with the charges specified in the fragment library. A 30 Å simulation sphere was used for both simulations in the bound and unbound states with inhibitors. The same sphere center was used as was described for the docking procedure, and it was again solvated with TIP3 water [23]. All atoms outside the 30 Å sphere were tightly restrained throughout the simulations. Water molecules at the surface of the sphere were subjected to radial and polarization constraints to mimic the properties of bulk water [24]. Before data collection, each simulation system was heated in a stepwise manner from 10 to 300 K with all solute heavy atoms subject to strong (10–25 kcal/mol Å^2^), harmonic positional restraints. Next, all simulations of the inhibitors in the bound state were equilibrated without restraints for 500 ps, followed by 250 ps of production phase MD. The final structure of the HRgpA:inhibitor complex was obtained after the equilibration step was used as a starting structure for the binding free energy calculation. To assess the problem of conformational sampling when simulating the inhibitors in the unbound state in water, 10 replicate water simulations of 5 ns each were performed for each inhibitor, with starting conformations generated by high temperature MD. For all production phase MD, a 1-fs time step was used along with the SHAKE procedure for all solvent bonds [25]. Non-bonded interactions across the simulation sphere boundary were excluded. A non-bonded cutoff of 10 Å was used, with electrostatic interactions outside the cutoff treated with the local reaction field multipole expansion [26], except for the inhibitor, which had no cutoff applied to any of its interactions.

### 3.3. Free Energy Calculation

Binding affinities were calculated using MD in combination with the Poisson Boltzmann Solvent Accessible Surface Area (PB-SASA) method [27–35], which uses simulations of the HRgpA:inhibitor complex to calculate the change in free energy associated with binding to the protein, according to the following equation: Δ*G*_bind_ = Δ*G*_MM_ + Δ*G*_solv_ −*T*Δ*S*, where Δ*G*_bind_ is the binding free energy, Δ*G*_MM_ is the molecular mechanical energy, Δ*G*_solv_ is the solvation energy, and *TΔS* is the entropy contribution. The molecular mechanical energy is calculated by the following equation: Δ*G*_MM_ = Δ*G*_elec_ + Δ*G*_vdW_, where Δ*G*_elec_ and Δ*G*_vdW_ represent electrostatic and van der Waals energies, respectively. In the PB-SASA method, the difference in interaction energies between the inhibitors and their surrounding residues are used to calculate the free energy of binding through the equation. MD simulations of the interacting molecules were conducted in water at *T* = 298 K and *P* = 1 atm using TIP3 water. The free energy of HRgpA:inhibitors association in aqueous solution (Δ*G*_solv_) can be divided into polar (electrostatic) (Δ*G*_elec_solv_) and non-polar (non-electrostatic) (Δ*G*_npol_solv_) terms [36]. The Poisson-Boltzmann equations have been shown to provide an accurate description of the electrostatic interactions between receptor and ligands. This approach was used in the present study to calculate electrostatic contributions to the binding free energy, while non-polar contributions were calculated using the SASA method [37–39]. Additionally, the transfer of a molecule from a phase of *ɛ* = 4 to a phase of *ɛ* = 78 justifies the application of the microscopic surface tension coefficient used in the SASA methods.

## 4. Conclusions

We have reported homology modeling, automated docking of benzamidine derivatives as inhibitors, and binding free energy calculations from molecular dynamics simulations of the gingipain R (HRgpA). The 2.0 Å crystal structure of the very closely related gingipain R (RgpB) [14] was used as a template for the homology modeling. Automated docking of eight benzamidine derivatives with known binding affinities was performed using the resulting three-dimensional model. For the top five docking solutions for each inhibitor, the structural and thermodynamic stabilities of the docked complexes were investigated further using the PB-SASA method in combination with MD simulations. The results from these simulations strongly suggest that all of the inhibitors bind to the RgpB in a manner similar to that of the benzamidine moiety of the inhibitors binding into HRgpA. Of the 800 suggested docking poses generated by automated docking, only a handful are positioned in a manner significantly different from the top-ranked poses, and the deviating poses are typically among the lowest ranked for that inhibitor. Furthermore, the inhibitors are found to be structurally stable in their docked positions during unrestrained MD simulations.

The bisbenzamidine inhibitors contain two aromatic rings linked by spacers with differing lengths and chemical characteristics. The bisbenzamidine inhibitors containing a urea moiety linking the two aromatic rings, such as compounds Bz1–4, were better inhibitors for both HRgpA and RgpB. Those bisbenzamidines which contain the less polar ether linker, such as Bz5–8, were less efficient inhibitors. Both isoforms of gingipain R showed a strong preference for the amidino group in the 4 position of the aromatic ring rather than at the 3 position [14]. Bisbenzamidine Inhibitors, which had a urea linker and the amidine substituent at the 3 position, were poorer inhibitors.

The accurate characterization of solvation effects is critical in the thermodynamic process of protein-ligand binding. The calculation of solvation free energies is a more tractable problem than predicting binding free energies, since the solvent molecules equilibrates more quickly around a ligand than around the binding site of the protein. The prediction of solvation free energies also provides a surrogate for the biologically relevant process of transferring a ligand from solution (high-dielectric environment) to the binding site of a protein (low-dielectric region) and, therefore, is an important step toward predicting accurate binding free energies. In the present study, the values of the free energy for HRgpA:bisbenzamidine complexes in TIP3 model are found to be in reasonably good agreement with the experimental values. From the present investigation, we conclude that the complexes in the TIP3 model are able to accurately describe the interaction between HRgpA and bisbenzamidine derivatives. But, further work on conducting the simulation in other water models such as TIP4P, SPC, and SPC/E models, could help to bring the accuracy level of binding free energy predictions to the point where they can provide substantial value.

In this work, the predicted binding modes of benzamidine derivatives to HRgpA are strongly supported by the following facts: The high sequence identity between RgpB and HRgpA in the binding region; a consensus docking pose for all inhibitors, which is also found to be stable during MD simulations; and an excellent correlation between observed and calculated binding affinities, where the latter were obtained from detailed all-atom energetic calculations.

Therefore, this model for benzamidine analog inhibitor binding to HRgpA, based on the RgpB template, is one of the few currently available examples of detailed three-dimensional models for how drug-like compounds interact with Arg-gingipain with the hemaglutaminine domain.

## Figures and Tables

**Table 1:** Observed binding free energies for benzamidine derivatives. The experimental values of HRgpA:inhibitors binding free energy. *K_i_* is the apparent inhibitory constant [14]. Δ*G*_exp_ is the calculated value of binding free energy according to the equation Δ*G*_exp_ = *RT* ln(*K_i_*) for *T* = 298 K. All energy values are reported in kcal/mol.

Name	Compounds	*K*_i_ (μM)	Δ*G*_exp_
Bz1	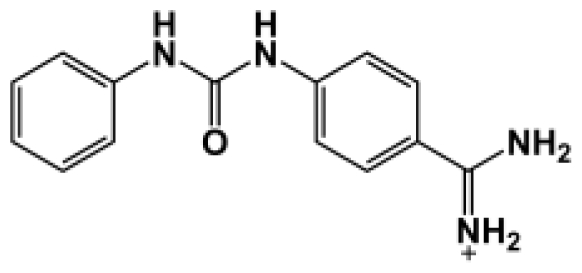	68.3	−5.676
Bz2	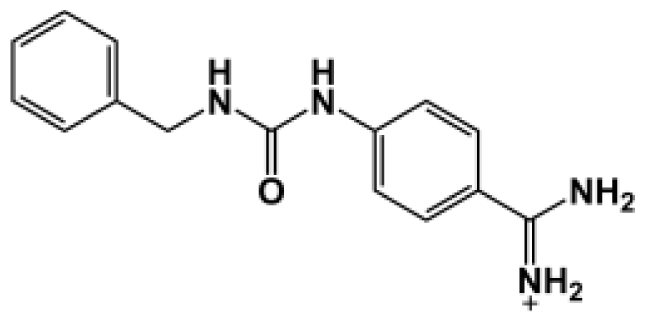	98.1	−5.461
Bz3	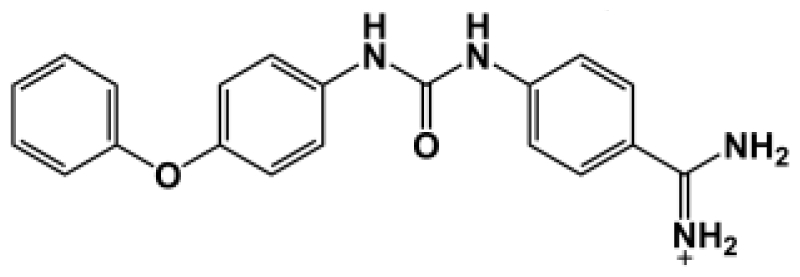	64.7	−5.708
Bz4	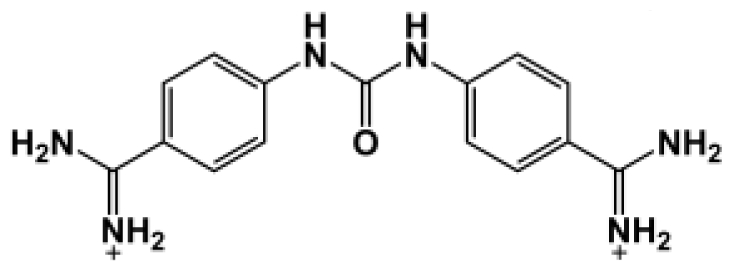	29.0	−6.183
Bz5	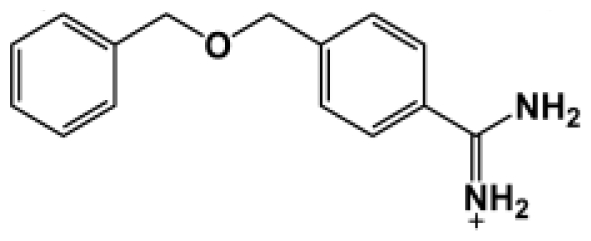	141.0	−5.247
Bz6	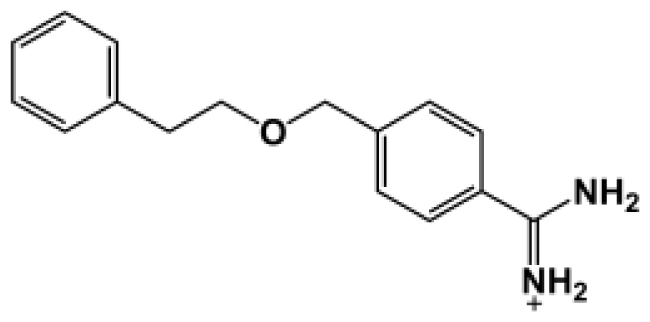	165.0	−5.154
Bz7	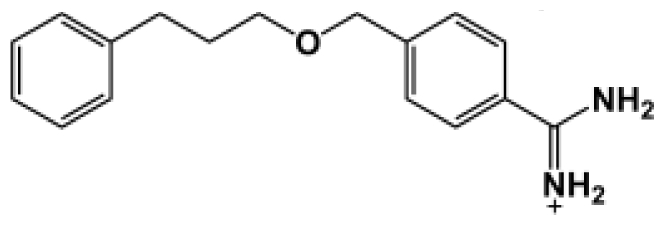	190.0	−5.070
Bz8	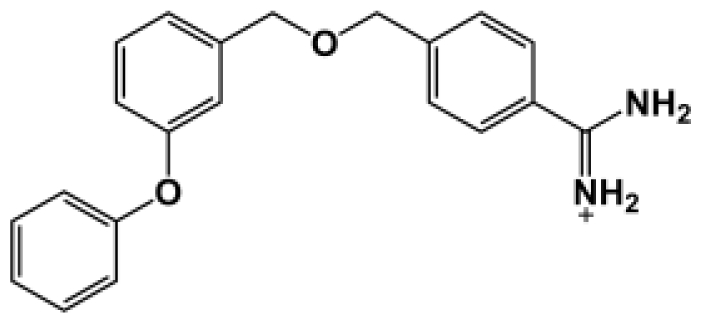	199.0	−5.043

**Table 2:** The calculated values for binding free energies of HRgpA complexed with benzamidine analogs. The electrostatic portion was calculated within the PB approach; the nonelectrostatic contribution was calculated using SASA methods. The binding portion is the sum of electrostatic and non-electrostatic contributions. All energy values are presented in kcal/mol. The uncertainties are the standard error of the mean calculated with 200 snapshots (50 snapshots for entropic calculations).

Name	Δ*G*_elec_	Δ*G*_vdW_	Δ*G*_nonp/sol_	Δ*G*_elec/sol_	−*T*Δ*S*	Δ*G*_bind_
Bz1	−25.89 ± 0.09	−13.88 ± 0.12	−3.42 ± 0.01	24.14 ± 0.01	12.36 ± 0.28	−6.69 ± 0.10
Bz2	−25.11 ± 0.01	−13.81 ± 0.12	−3.45 ± 0.01	24.24 ± 0.03	12.61 ± 0.54	−5.52 ± 0.14
Bz3	−25.21 ± 0.11	−14.33 ± 0.12	−3.31 ± 0.01	23.23 ± 0.01	12.79 ± 0.31	−6.83 ± 0.11
Bz4	−26.21 ± 0.10	−15.02 ± 0.13	−3.28 ± 0.01	23.05 ± 0.04	12.38 ± 0.63	−9.08 ± 0.18
Bz5	−24.26 ± 0.11	−13.52 ± 0.07	−3.34 ± 0.01	23.51 ± 0.02	13.63 ± 0.55	−4.68 ± 0.15
Bz6	−26.03 ± 0.10	−13.37 ± 0.16	−3.18 ± 0.01	24.31 ± 0.02	14.25 ± 0.39	−4.02 ± 0.14
Bz7	−25.55 ± 0.08	−13.78 ± 0.11	−3.45 ± 0.01	24.61 ± 0.05	14.57 ± 0.71	−3.60 ± 0.12
Bz8	−25.16 ± 0.09	−13.03 ± 0.13	−3.04 ± 0.01	24.15 ± 0.03	13.69 ± 0.59	−3.39 ± 0.17
